# Intrapartum Asphyxiated Newborns Without Fetal Heart Rate and Cord Blood Gases Abnormalities: Two Case Reports of Shoulder Dystocia to Reflect Upon

**DOI:** 10.3389/fped.2020.570332

**Published:** 2020-10-27

**Authors:** Gina Ancora, Claudio Meloni, Silvia Soffritti, Fabrizio Sandri, Emanuela Ferretti

**Affiliations:** ^1^Neonatal Intensive Care Unit, Department of Maternal, Infant and Adolescent Health, Infermi Hospital, Azienda Unità Sanitaria Locale Romagna, Rimini, Italy; ^2^Obstetrics and Gynecology Unit, Department of Maternal, Infant and Adolescent Health, Infermi Hospital, Azienda Unità Sanitaria Locale Romagna, Rimini, Italy; ^3^Neonatal Intensive Care Unit, Department of Maternal and Infant Health, Maggiore Hospital, Bologna, Italy; ^4^Division of Neonatology, Department of Pediatrics, Children's Hospital of Eastern Ontario, The Ottawa General Hospital, University of Ottawa, Ottawa, ON, Canada

**Keywords:** shoulder dystocia, asphyxia, hypoxic ischemic encephalopathy, cardiac asystole theory, intact cord resuscitation, hypovolemia

## Abstract

Our report covers two cases of severe hypoxic-ischemic encephalopathy in newborns whose birth was complicated by shoulder dystocia. In both cases, there were inconsistencies observed among cardiotocographic traces, baby's clinical conditions at birth, and umbilical cord blood gases. Namely, normal cardiotocographic monitoring and cord pH > 7, in spite of the fact that the newborns were severely depressed at birth and their blood gases evaluated within 1 h from birth showed a severe metabolic acidosis. Moreover, one of the two newborns displayed moderately low hemoglobin levels. Metabolic and infectious causes were ruled out. Both newborns developed severe hypoxic-ischemic encephalopathy and received therapeutic hypothermia for 72 h. Both survived, one with a severe dystonic cerebral palsy whereas the other developed only a mild developmental delay in language. Cardiac asystole theory could explain these two cases, reinforcing the need for specific resuscitation guidelines for infants experiencing a birth complicated by shoulder dystocia.

## Introduction

Shoulder dystocia is a severe condition capable of posing a risk to the health of the newborn by increasing the odds of perinatal asphyxia, hypoxic-ischemic encephalopathy, perinatal mortality, and brachial plexus paralysis (1).

Asphyxia following shoulder dystocia is sometimes very severe, and perinatal mortality has been reported in spite of a remarkably short head-body delivery interval (2). In these dramatic circumstances, inconsistencies can be detected among neonatal clinical status, cardiotocographic findings, and cord blood gases values (3–5), possibly increasing litigation between healthcare professionals and parents. Here we report two cases of infants whose birth was complicated by shoulder dystocia, who were severely depressed at birth despite the lack of fetal heart rate (FHR) anomalies during labor or umbilical-cord gas anomalies at birth, and despite appropriate obstetric and neonatological care; thus, reinforcing the soundness of the cardiac asystole hypothesis in these cases (3).

### Case 1

The firstborn male of a healthy G1, P0, 21-year old mom, was born at 41+1 weeks gestation, birth weight 3,700 g, by vaginal delivery after spontaneous rupture of membrane (SROM) of 15 h; two vacuum tractions were performed because of a prolonged active second stage of labor. Cardiotocographic monitoring was normal for the expulsive period. After the extraction of the fetal head, a maneuver of digital hooking (Bourgeois maneuver) was performed for the disengagement of the shoulders. The delay between the extraction of the fetal head and the disengagement of the shoulders was 2 min. There was a presence of thick meconium-stained amniotic fluid after the fetal extraction. At birth, the umbilical cord was immediately clamped. The baby was in cardiac asystole at auscultation, without spontaneous respiratory efforts, pale, and hypotonic. Cardiopulmonary resuscitation was started and continued according to the neonatal resuscitation program (NRP) (6). A pulse oximeter was immediately positioned, followed by probe-ECG monitoring. Resuscitation included positive-pressure ventilation, chest compressions, intubation, and epinephrine. At 7 min of life, the heart rate was >100 beats per minute (bpm). The baby showed severe hypotonia and passive hypothermia was started at about 10 min of life. He was transferred to the neonatal intensive care unit (NICU), mechanically ventilated with a fraction of inspired oxygen (FiO_2_) 0.3. His Apgar scores were 0, 0, 5 at 1, 5, and 10 min, respectively. The cord blood gas analysis revealed an arterial pH 7.16, pCO_2_ 58 mmHg (kPa 7.7), base excess (BE) −8.4 mmol/l, lactate 10.2 mmol/l; the pH from the venous sample was 7.26, pCO_2_ 42 mmHg (kPa 5.6), BE −7.5 mmol/l, lactate 8.8 mmol/l.

Upon arrival in the NICU, his umbilical vein was catheterized, and blood work was done. Capillary gas performed on the neonate at about 30 min of life showed a pH 6.8, pCO_2_ 83 mmHg (kPa 11.0), BE −21 mmol/l, lactate 14 mmol/l, and glucose 4.7 mmol/l. A neurologic exam showed moderate hypoxic-ischemic encephalopathy. Amplitude-integrated electroencephalography (aEEG) monitoring showed a burst suppression type trace. The arterial gas at 1 h of life showed pH 7.01, pCO_2_ 58 (kPa 7.7), BE −21 mmol/l, and lactate 18 mmol/l. Hemoglobin at 14 h of life was 12.1 g/dl. Active hypothermic treatment was started and maintained for 72 h. Because of clinical suspicion of seizures, the baby was loaded with Phenobarbital but not maintained due to a lack of electroencephalographic abnormalities and normalization of the clinical picture. At about 4 h after birth, as spontaneous respiration improved, the newborn was extubated and gradually weaned off any ventilatory support. At 13 days of life (DOL), the overall neurologic exam showed a favorable evolution. The otoacoustic emission test results were normal, bilaterally. He was discharged on 14 DOL. His first head magnetic resonance imaging (MRI), done at 1 month of life, was normal. Follow-up at 30 months of age showed only a mild developmental delay in language and he required follow-up sessions with a speech therapist.

### Case 2

A female newborn was born at 41+6 weeks gestation, from a 32-year old, G1, P0 mom. The pregnancy was spontaneous and without complications. The mother had been admitted the day before to the hospital ward and labor was induced with prostaglandins for post-term pregnancy. The spontaneous rupture of the membranes occurred 13 h before delivery. The amniotic fluid was slightly stained with meconium. At 7 cm of cervical dilatation, infusion with oxytocin was started. Continuous cardiotocographic monitoring showed no abnormalities. Her birth was assisted by a midwife and a gynecologist. When the head came out with difficulty, the anterior part of the newborn's shoulder was trapped under the pubic symphysis. The doctor performed three maneuvers in succession (Mc Roberts, Woods, and Jacquemier) obtaining the disengagement of the shoulders; the head-body delivery interval was 3 min. At birth, the umbilical cord was immediately clamped. The arterial and vein cord pH were 7.09 (lactate 10.3 mmol/l) and 7.17 (lactate 9.3 mmol/l), respectively. The newborn was apneic, asystolic at auscultation, atonic, and areflexic. Resuscitation was immediately started and continued up to endotracheal intubation and chest compressions. Pulse oximetry and ECG confirmed persistent asystole, therefore, one dose of epinephrine, followed by a normal saline bolus was administered via the cannulated umbilical vein. Heart rate was detected at 13 min of life and spontaneous respiratory activity resumed 40 min after birth. Apgar score was 0, 0, 0, 3 at 1, 5, 10, and 15 min.

The newborn was then transferred to NICU and mechanically ventilated. The temperature at admission, after passive cooling initiated in the delivery room, was 34°C. At 1 h from birth, the venous gas analysis showed a pH 7.10, pCO_2_ 29.1 mmHg (kPa 3.9) BE −19 mmol/l, HCO_3_ 10.5 mmol/l, and lactate 9.8 mmol/l. Hemoglobin values at birth and at 2 days of life were 15.2 and 14.9 g/dl, respectively. The neurologic exam showed a severe hypoxic-ischemic encephalopathy. Since the aEEG was profoundly abnormal, systemic hypothermic treatment was started and continued for 72 h. She received Phenobarbital to treat seizures. At DOL 5, her head MRI confirmed a hypoxic-ischemic insult mainly at the level of the deep gray matter. On DOL 6 she was extubated to nasal continuous positive airways pressure, and then to humidified high flow nasal cannulae. At the 2-years follow-up, the baby showed severe dystonic cerebral palsy and neurosensorial deficits.

## Discussion

The two cases described in the present study are similar to three other cases reported in literature (4, 5) where shoulder dystocia, resolved in <5 min, was associated with normal FHR tracings, arterial cord pH > 7.0, severe neonatal depression at birth, and early severe neonatal metabolic acidosis (7, 8). The same discrepancy among normal FHR tracings, normal cord blood-gas values, and severe neonatal acidosis has been reported in two other severely depressed cases in which the time from delivery of the head to the delivery of the body was estimated to be between 5 and 10 min (3).

A plausible pathogenetic mechanism, called *Cardiac Asystole Theory* (3), has been already postulated for these cases and could also be applied to our newborns. In fact, in the case of shoulder dystocia, compression of the umbilical cord between the fetus and the birth canal might occur.

In this specific circumstance, pH and blood gases could remain stable up to 1 h after the obstruction; whereas, lactate might increase due to anaerobic metabolism in cord erythrocytes, leucocytes, endothelial cells, and in the placenta, as documented in studies conducted on clamped and unclamped cord vessels after placental delivery (9). This can explain our findings of abnormally elevated lactate with an almost normal pH in cord blood.

In these cases, the fetal status can be only documented by a blood gas analysis obtained directly from the newborn after birth as reported in the literature and confirmed by our cases. Sampling performed directly on our newborn patients at 1 h of life, confirmed severe metabolic acidosis.

Complete cord obstruction also induces blood sequestration within the placenta and leads to fetal hypovolemia. In shoulder dystocia, the birth canal would function as an “anti-shock garment” capable of maintaining high peripheral pressures even in the presence of low circulating volume, thus allowing good central perfusion and a normal pulse (3). This mechanism could explain the normality of FHR in our two cases. The sudden release of pressure occurring at the time the fetus comes out of the birth canal would cause a rapid redistribution of blood into the peripheral circulation, cardiac arrest for severe central hypoperfusion, and hypovolemic shock.

In the present report, both newborns appeared extremely depressed and pale at birth; nevertheless, cord milking or “early” volume infusion were not used because blood loss was not suspected. Indeed, it has not been definitively demonstrated that shoulder dystocia can be associated with volume depletion in the newborn. However, in light of the discussed pathogenetic postulate, clinicians facing a severely depressed newborn with shoulder dystocia, with normal FHR, and high cord lactate levels—but without evidence of pathological acidaemia at cord blood pH (7, 8)- should suspect a complete cord obstruction with blood sequestration within the placenta. In these cases, clinicians should consider the potential role of early fluid resuscitation. Moreover, adopting resuscitation with an intact umbilical cord or after delayed cord clamping/cord milking might be useful in cases of shoulder dystocia, although it will take concentrated effort and teamwork by midwifery, obstetrics, neonatologist, and nursing (10). Nowadays, resuscitation with an intact cord, or cord milking, can be done thanks to the use of a specific mobile trolley that allows newborn resuscitation at the mother's bedside (11). Resuscitation with an intact cord, whose objective is to establish adequate ventilation before proceeding with the clamping of the umbilical cord, favors the passage of 15–30 ml/kg of blood to the newborn, the equivalent of a transfusion (12). In the presence of bradycardia or cardiac arrest, this passage does not occur, and the milking of the umbilical cord may be useful to facilitate the passage of blood from the placenta to the newborn. Despite the large body of evidence demonstrating that delayed cord clamping has benefits for term and preterm infants, this approach still has organizational and logistical limitations. In units where resuscitation with an intact cord has been implemented, maybe in the context of clinical trials, it may be easier to overcome logistical issues and extend this practice in an emergency, such as shoulder dystocia.

The NRP does not recommend delayed cord clamping in asphyxiated newborns (6). However, recent studies suggest that in term infants, resuscitation with an intact umbilical cord is associated with a better recovery than routine resuscitation and that umbilical cord milking appears to be a safe therapy when resuscitation is needed (13–15).

Sharing knowledge on physiopathological changes occurring in newborns experiencing shoulder dystocia among healthcare professionals who provide care around the time of birth (neonatologists, obstetricians and gynecologists, anesthesiologists, pediatricians), could help to improve the understanding of these dramatic cases. Dissemination of this knowledge is also useful to improve resuscitation reaction of the asphyxiated newborn whose birth is complicated by shoulder dystocia. In these cases, the first importance is to avoid unjustified delayed volume replacement during resuscitation. Secondly, it could be useful to provide resuscitation at the mother's bedside, while the obstetrician provides cord milking.

Future efforts need to be undertaken to prospectively gather data to deepen the knowledge and to refine the therapeutic approach to the potentially harmful condition of shoulder dystocia.

## Conclusions

The combined findings of normal FHR, almost normal cord pH and blood gases, elevated cord lactate, very low pH on blood gas analysis obtained within 1 h of life and severe cardiorespiratory depression in newborns with shoulder dystocia, justify the suspicion of complete cord obstruction and blood sequestration within the placenta. These newborns should be regarded as volume-depleted and resuscitated with volume expansion.

This report raises awareness to clinicians resuscitating newborns with shoulder dystocia about the underlying mechanism of asphyxia in this condition. Future efforts toward resuscitation guidelines are needed to refine the therapeutic approach facing this potentially harmful condition.

## Data Availability Statement

The raw data supporting the conclusions of this article will be made available by the authors, without undue reservation.

## Ethics Statement

Ethical review and approval was not required for the study on human participants in accordance with the local legislation and institutional requirements. Written informed consent to participate in this study was provided by the participants' legal guardian/next of kin. Written informed consent was obtained from the individual(s), and minor(s)' legal guardian/next of kin, for the publication of any potentially identifiable images or data included in this article.

## Author Contributions

GA and CM conceived the presented idea. GA, CM, SS, and FS reviewed the case reports. EF supervised the findings of this work. All authors discussed the results and contributed to the final manuscript.

## Conflict of Interest

The authors declare that the research was conducted in the absence of any commercial or financial relationships that could be construed as a potential conflict of interest.
